# The Type of Bariatric Surgery Impacts the Risk of Acute Pancreatitis: A Nationwide Study

**DOI:** 10.1038/s41424-018-0045-0

**Published:** 2018-09-12

**Authors:** Hisham Hussan, Emmanuel Ugbarugba, Kyle Porter, Sabrena Noria, Bradley Needleman, Steven K. Clinton, Darwin L. Conwell, Somashekar G. Krishna

**Affiliations:** 1 Obesity and Bariatric Endoscopy Section, Division of Gastroenterology, Hepatology, & Nutrition, Department of Internal Medicine, The Ohio State University Wexner Medical Center, Columbus, OH USA; 2 Division of Hospital Medicine, Department of Internal Medicine, The Ohio State University Wexner Medical Center, Columbus, OH USA; 3 Center for Biostatistics, Department of Biomedical Informatics, The Ohio State University, Columbus, OH USA; 4 Center for Minimally Invasive Surgery, Department of General Surgery, The Ohio State University Wexner Medical Center, Columbus, OH USA; 5 Division of Medical Oncology, Department of Internal Medicine, The Ohio State University Wexner Medical Center, Columbus, OH USA; 6 Division of Gastroenterology, Hepatology, & Nutrition, Department of Internal Medicine, The Ohio State University Wexner Medical Center, Columbus, OH USA; 7 Comprehensive Cancer Center, The Ohio State University, Columbus, OH USA

## Abstract

**Objective:**

We investigated whether vertical sleeve gastrectomy (VSG) and Roux-en-Y gastric bypass surgery (RYGB) have a differential impact on post-operative risk of acute pancreatitis (AP).

**Methods:**

This retrospective study uses the 2012–2014 National Readmission Database. We compared morbidly obese patients who underwent VSG (*n* = 205,251), RYGB (*n* = 169,973), and hernia repair (HR) control (*n* = 16,845). Our main outcome was rates of AP within 6 months post- vs. 6 months pre-surgery in VSG, RYGB, and HR. We also investigated risk factors and outcomes of AP after bariatric surgery.

**Results:**

The rates of AP increased post- vs. pre-VSG (0.21% vs. 0.04%; adjusted odds ratio [aOR] = 5.16, *P* < 0.05) and RYGB (0.17% vs. 0.07%; aOR = 2.26, *P* < 0.05) but not post-HR. VSG was associated with a significantly greater increase in AP risk compared to RYGB (aOR = 2.28; 95% CI: 1.10, 4.73). Furthermore, when compared to HR controls, only VSG was associated with a higher AP risk (aOR = 7.58; 95% CI: 2.09, 27.58). Developing AP within 6 months following bariatric surgery was mainly associated with younger age (18–29 years old: aOR = 3.76 for VSG and aOR: 6.40 for RYGB, *P* < 0.05) and gallstones (aOR = 85.1 for VSG and aOR = 46 for RYGB, *P* < 0.05). No patients developed “severe AP” following bariatric surgery.

**Conclusions:**

More patients develop AP within 6 months after VSG compared to RYGB and controls. This risk is highest for younger patients and those with gallstones. Prospective studies examining mechanisms and prevention are warranted.

## Introduction

Acute pancreatitis (AP) is a leading gastrointestinal cause of hospital admissions in the U.S. with over 275,170 AP admissions in 2012^1^. Furthermore, the annual rates of AP admissions are rising with a resultant staggering economic burden estimated at $2.6 billion per year for inpatient costs alone^1–3^. Obesity, defined as a body mass index (BMI) ≥ 30 kg/m2, also affects nearly 36% of U.S. adults, with no decrease in obesity prevalence according to recent national surveys^4–6^. The increasing obesity rates are in parallel with AP, possibly due to an increased gallstone risk in obese individuals^7–10^. Furthermore, obesity is an independent predictor of severity, end-organ failure, and mortality in patients admitted with AP^3^.

Bariatric surgery remains a safe and effective long-term weight loss treatment for morbidly obese patients^11–14^. Vertical sleeve gastrectomy (VSG) has been the most common bariatric surgery since 2013, followed by Roux-en-Y (RYGB)^15^. Both VSG and RYGB improve the comorbidities, metabolic profile, and the inflammatory state seen in obesity^13,16–20^. Consequently, data indicate that a history of bariatric surgery is associated with improved AP outcomes when compared to morbid obesity^21^. Alternatively, bariatric surgery is associated with increased risk of gallstone disease and minimal data exists on differential impact of bariatric surgery type on risk of AP^22,23^. Single-center studies reported high rates of AP after RYGB and VSG, ranging between 0.2 and 1.04%, compared to previously reported annual AP incidence rates of 0.013–0.045%^22–33^. No published research has directly compared post-operative AP rates after VSG or RYGB to a control surgery or examined national estimates. We hypothesize that the bariatric surgery type has a differential impact on risk of AP, and the confirmation of such an association would contribute to better prognostication of bariatric patients at risk of AP. To test this hypothesis, we aimed to quantify the impact of VSG and RYGB surgeries on AP rates compared to a control procedure, such as surgical hernia repair, and determine risk factors and outcomes of AP after RYGB and VSG.

## Method

### The National Readmission Database

All data were extracted from the 2012–2014 The National Readmission Database (NRD). The Healthcare Cost and Utilization Project’s (HCUP) NRD is a unique database of hospital inpatient stays for all payer types that can be used to examine national estimates of readmission rates. The database is drawn from HCUP State Inpatient Databases containing verified patient linkage numbers that can be used to track a person across hospitals within a state while adhering to strict privacy guidelines^34^. Thus, the dataset captures admissions to hospitals other than the hospital where the surgery was performed. The NRD is a stratified, single-stage cluster sample of hospital discharges with weights that can be used to provide nationally-representative estimates. Weighted NRD admissions represent approximately 36 million discharges every year in the United States. It includes 21 HCUP Partner States and accounts for 49.1% of all U.S. hospitalizations. The data contained within the NRD database are neither identifiable nor private and hence do not meet the federal definition of “human subject”. Our study was therefore exempt from Institutional Review Board oversight.

### Study cohort

**Fig. 1:**
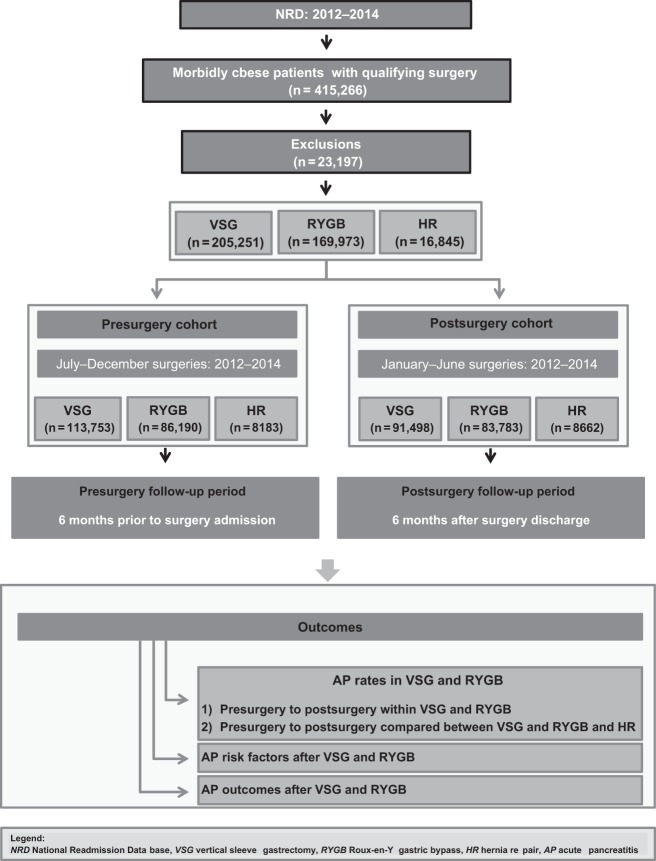
Flowchart showing study design and outcomes

The study design is shown in Fig. 1. The NRD was queried using ICD-9-CM codes to identify index admissions of morbidly obese patients (BMI ≥ 35 kg/m^2^) who underwent elective laparoscopic RYGB, VSG, or HR^35^ (Table 4S). The ICD-9-CM code for AP has been validated and used in prior studies^3,21,36^. We chose HR (ventral, umbilical, inguinal, or diaphragmatic) patients as surgical controls since HR could mimic a sham surgery without bowel alteration or the post-surgical weight loss observed after bariatric surgery^35,37–40^. Patients were excluded if they met any of the following conditions: (a) age < 18 years; (b) pregnancy; (c) abdominal malignancy;^41,42^ (d) hernia surgery performed for indications other than hernia treatment; (e) hernia with gangrene or an obstruction; (f) previous bariatric surgery; (g) diagnosis of chronic pancreatitis or pancreatic cysts; (h) open, emergent surgery or multiple surgeries;^43^ (i) mortality or AP on index surgery admission; or (j) the admission length of stay was not reported. In order to compare pre- to post-surgery AP rates, we elected to divide all three groups (VSG, RYGB, and HR) into two cohorts with equal follow-up periods as in Fig. 1: (1) The pre-surgery cohorts included patients who had surgeries with discharge months between July and December of 2012–2014. In this cohort, AP rates were investigated within six months prior to the surgery admission date. (2) The post-surgery cohorts included patients who had surgeries with discharges between January and June of 2012–2014. This cohort was followed for six months post-surgery discharge.

### Outcomes

Outcomes of interest included the following (Fig. 1): (1) pre- to post-surgery AP rates for VSG, RYGB, and HR; (2) a comparison of pre- to post-surgery AP rates among these patients; (3) risk factors associated with AP in the post-surgical period for VSG and RYGB patients; and (4) AP outcomes after RYGB and VSG (i.e., severity, interventions, surgeries, subsequent cholecystectomies, and AP readmissions). Risk factors or outcomes of AP after HR were not studied due a low rate of AP events in these patients.

### Statistical Analysis

Analyses were performed using SURVEY procedures in SAS version 9.4 (SAS Institute Inc., Cary, NC). Multivariable weighted logistic regression was used to compare the odds of AP admission in the pre- vs. post-surgery study periods for each type of surgery. Interactions were utilized to compare pre- vs. post-surgery odds ratios between each pair of surgeries; for example, pre- vs. post-surgery in VSG compared to the same in controls. The primary model adjusted for age, gender, and Elixhauser comorbidities (27 disease states excluding obesity) created by the Agency for Healthcare Research and Quality (AHRQ) and described by Elixhauser et al^44^. In a sensitivity analysis, AP risk factors were also adjusted for such as alcohol use, gallstones (cholelithiasis or choledocholithiasis), and prior cholecystectomy. Alcohol use and gallstones were defined as presence of their respective ICD-9-CM codes during the follow-up period including the index surgery and AP admission. Prior cholecystectomy was defined as presence of corresponding codes during the study period and before the AP episode. Risk factors for AP admission within 6 months post-surgery were assessed by weighted logistic regression for VSG and RYGB separately. Univariable models were fit using all candidate risk factors followed by a multivariable model, which included all risk factors with *p* < 0.1 in the univariable analysis. A gallstone by cholecystectomy interaction was added to each model. Etiologies and procedures for AP admissions within 6 months post-surgery were summarized for VSG and RYGB. The only variables containing missing values were primary payer (*n* = 215), which was assigned to the “other” category; missing patient-income data were assigned to a separate category. All statistical tests were evaluated at the *α* = 0.05 significance level. No adjustments were made for multiple comparisons.

## Results

### Patients’ characteristics

The pre- and post- surgery cohorts were similar, but cholecystectomy rates were lower in the pre-surgery cohort compared to post-surgery. (Table 1). HR patients were older than RYGB and VSG but with a similar gender distribution. Most patients had less than three comorbidities at the time of surgery. Further analysis shows similar comorbidities between VSG, RYGB, and HR, although RYGB patients had slightly higher rates of diabetes and liver disease at index surgery admission (Table 1S). The predominant payer source was private insurance, but the bariatric groups had a higher percentage of private pay insurance and lower Medicare payer than HR patients. Compared to RYGB and VSG, HR patients had higher rates of alcohol use, gallstones, and prior cholecystectomy. The hospital characteristics were similar for all cohorts.

**Table 1:** Patient and hospital characteristics

Study cohort	Pre-surgery cohort			Post-surgery cohort		
Surgery type	RYGB (*n* = 86,190)	VSG (*n* = 113,753)	HR (*n* = 8,183)	RYGB (*n* = 83,783)	VSG (*n* = 91,498)	HR (*n* = 8,662)
Age, mean (SE)	45.4 (0.2)	44.4 (0.1)	54.1 (0.3)	45.1 (0.2)	44.0 (0.1)	53.4 (0.3)
Age range						
18–29	8,824 (10.2%)	12,417 (10.9%)	241 (2.9%)	8,931 (10.7%)	10,536 (11.5%)	333 (3.8%)
30–49	44,347 (51.5%)	61,982 (54.5%)	2,782 (34.0%)	43,248 (51.6%)	50,669 (55.4%)	2,992 (34.5%)
≥50	33,019 (38.3%)	39,355 (34.6%)	5,160 (63.1%)	31,605 (37.7%)	30,293 (33.1%)	5,336 (61.6%)
Sex						
Male	18,207 (21.1%)	24,651 (21.7%)	1,883 (23.0%)	17,766 (21.2%)	20,198 (22.1%)	2,070 (23.9%)
Female	67,982 (78.9%)	89,102 (78.3%)	6,300 (77.0%)	66,017 (78.8%)	71,301 (77.9%)	6,592 (76.1%)
Index length of stay, days, median (IQR)	1.5 (1–1.9)	1.2 (0.7–1.8)	1.6 (0.7–3)	1.5 (1.1–1.9)	1.3 (0.7–1.8)	1.4 (0.6–2.7)
Index length of stay, days						
1–2	68,851 (79.9%)	98,565 (86.6%)	4,981 (60.9%)	66,075 (78.9%)	78,866 (86.2%)	5,625 (64.9%)
≥3	17,338 (20.1%)	15,188 (13.4%)	3,202 (39.1%)	17,709 (21.1%)	12,632 (13.8%)	3,037 (35.1%)
Elixhauser index (minus obesity)						
0	11,893 (13.8%)	23,579 (20.7%)	1,273 (15.6%)	12,099 (14.4%)	19,490 (21.3%)	1,388 (16.0%)
1–2	47,352 (54.9%)	64,928 (57.1%)	4,376 (53.5%)	45,978 (54.9%)	52,657 (57.5%)	4,760 (55.0%)
3–4	23,268 (27.0%)	22,289 (19.6%)	2,046 (25.0%)	22,283 (26.6%)	17,244 (18.8%)	2,065 (23.8%)
≥ 5	3,676 (4.3%)	2,956 (2.6%)	488 (6.0%)	3,424 (4.1%)	2,108 (2.3%)	449 (5.2%)
Primary payer						
Medicare	16,253 (18.9%)	13,879 (12.2%)	2,894 (35.4%)	16,316 (19.5%)	10,762 (11.8%)	3,094 (35.7%)
Medicaid	12,244 (14.2%)	12,173 (10.7%)	848 (10.4%)	12,519 (14.9%)	9,915 (10.8%)	864 (10.0%)
Private insurance	51,089 (59.3%)	79,717 (70.1%)	3,847 (47.1%)	48,587 (58.0%)	64,173 (70.1%)	3,988 (46.1%)
Self-pay	2,021 (2.3%)	5,672 (5.0%)	250 (3.1%)	2,110 (2.5%)	5,122 (5.6%)	273 (3.2%)
Other	4,572 (5.3%)	2,309 (2.0%)	329 (4.0%)	4,235 (5.1%)	1,526 (1.7%)	440 (5.1%)
Income quartile						
Quartile 1	20,377 (23.7%)	23,997 (21.1%)	1,998 (24.4%)	19,591 (23.4%)	19,037 (20.8%)	2,182 (25.2%)
Quartile 2	23,409 (27.2%)	28,031 (24.7%)	2,197 (26.9%)	22,787 (27.2%)	22,135 (24.2%)	2,144 (24.8%)
Quartile 3	23,503 (27.3%)	29,988 (26.4%)	2,108 (25.8%)	22,513 (26.9%)	23,996 (26.2%)	2,279 (26.3%)
Quartile 4	17,700 (20.6%)	30,227 (26.6%)	1,759 (21.5%)	17,810 (21.3%)	25,178 (27.5%)	1,923 (22.2%)
Missing data	1,141 (1.3%)	1,434 (1.3%)	118 (1.4%)	1082 (1.3%)	1,151 (1.3%)	132 (1.5%)
Hospital type						
Urban non-teaching	26,229 (30.4%)	37,913 (33.3%)	2,507 (30.6%)	26,414 (31.5%)	29,735 (32.5%)	2,764 (31.9%)
Urban teaching	57,840 (67.1%)	72,772 (64.0%)	5,138 (62.8%)	55,375 (66.1%)	59,616 (65.2%)	5,426 (62.6%)
Rural	2,121 (2.5%)	3,067 (2.7%)	538 (6.6%)	1,994 (2.4%)	2,148 (2.3%)	472 (5.5%)
Hospital bedsize						
Small	14,319 (16.6%)	18,761 (16.5%)	1,519 (18.6%)	13,031 (15.6%)	14,956 (16.3%)	1,355 (15.6%)
Medium	20,811 (24.1%)	29,660 (26.1%)	1,822 (22.3%)	20,789 (24.8%)	24,203 (26.5%)	2,132 (24.6%)
Large	51,060 (59.2%)	65,332 (57.4%)	4,842 (59.2%)	49,963 (59.6%)	52,339 (57.2%)	5,175 (59.7%)
Alcohol use	236 (0.3%)	370 (0.3%)	79 (1.0%)	332 (0.4%)	292 (0.3%)	74 (0.9%)
Gallstones	2,888 (3.4%)	2,902 (2.6%)	557 (6.8%)	3,419 (4.1%)	2,592 (2.8%)	542 (6.3%)
Prior cholecystectomy	191 (0.2%)	224 (0.2%)	36 (0.4%)	5,150 (6.1%)	4,347 (4.8%)	935 (10.8%)

### Impact of RYGB and VSG on AP rates compared to HR controls

The incidence of pre- and post-surgery AP is illustrated in Table 1. HR had the highest rate of pre-surgery AP (0.17%) while VSG had the highest post-surgery AP rate (0.21%). Most post-surgery AP admissions occurred within 30 days after VSG (58.3%) and RYGB (48.2%). After adjusting for confounding variables, VSG had the highest increased odds ratios for pre- to post-surgery AP (aOR = 5.16; 95%CI: 3.11, 8.56; *P* < 0.001), followed by RYGB (aOR = 2.26; CI; 1.33, 3.87, *P* = 0.003), and AP risk did not increase after HR (Table 2). VSG was associated with a significant increase in pre- to post-surgery AP risk when compared to each RYGB and HR on multivariable analyses (VSG vs. RYGB: adjusted odds ratio [aOR] = 2.28; 95% CI: 1.10, 4.73; VSG vs. HR: aOR = 7.58; 95% CI: 2.09, 27.58;) as seen in Table 3. Conversely, RYGB trended towards increased pre- to post-surgery AP risk when compared to HR although it was not significant (aOR = 3.33; 95% CI: 0.91, 12.18; *P* = 0.07). A sensitivity analysis also adjusted for gallstones, alcohol use, and prior cholecystectomy. The increased AP risk within surgery became more pronounced although it remained nonsignificant for HR (Table 2). Furthermore, the pre- to post-surgery AP risk remained elevated in VSG compared to HR and RYGB, which is presented in Table 3 (VSG vs. HR: aOR = 4.74; 95% CI:1.27, 17.64; VSG vs. RYGB: aOR = 2.31; 95% CI: 1.11, 4.81). RYGB was not associated with an increased pre- to post-surgery AP risk compared to HR (aOR = 2.05; 95% CI: 0.55, 7.65).

**Table 2:** Rates and comparison of six months pre- and post-surgery AP within surgery groups

Surgery type	Six months post-surgery	Six months post-surgery	Post vs. Pre within surgery, odds ratio (95% confidence interval), *p*-value		
			Univariable	Multivariable^a^	Sensitivity analysis multivariable^b^
VSG	47 (0.04%)	196 (0.21%)	5.21 (3.15, 8.64), *P* < 0.001	5.16 (3.11, 8.56), *P* < 0.001	7.27 (4.26, 12.38), *P* < 0.001
RYGB	62 (0.07%)	138 (0.17%)	2.29 (1.34, 3.90), *P* = 0.002	2.26 (1.33, 3.87), *P* = 0.003	3.15 (1.76, 5.63), *P* < 0.001
HR	14 (0.17%)	^c^ (≤0.12%)	0.67 (0.20, 2.19), *P* = 0.50	0.68 (0.21, 2.23), *P* = 0.68	1.53 (0.45, 5.26), *P* = 0.50

*VSG* vertical sleeve gastrectomy, *RYGB* Roux-en-Y gastric bypass, *HR h*ernia repair, AP acute pancreatitis

^a^Model covariates include age, sex, index admission length of stay, and Elixhauser comorbidities (minus obesity)

^b^Sensitivity analysis model covariates include age, sex, index admission length of stay, Elixhauser comorbidities (minus obesity), alcohol use, gallstones, and prior cholecystectomy

^c^The cell’s value is not displayed. As per data agreements with AHRQ, researchers cannot report any statistics where the number of observations in any given cell of analyzed data is ≤10

**Table 3:** Comparison of AP risk within six months pre- and post-surgery among RYGB, VSG, and HR controls

Comparison	Univariable odds ratio (95% CI), *P*	Multivariable ^a^ odds ratio (95% CI), *P*	Sensitivity analysis Multivariable ^b^ odds ratio (95% CI), *P*
Post vs. Pre in VSG compared to Post vs. Pre in RYGB	2.28 (1.10, 4.71), *P* = 0.03	2.28 (1.10, 4.73), *P* = 0.03	2.31 (1.11, 4.81), *P* = 0.03
Post vs. Pre in VSG compared to Post vs. Pre in HR	7.81 (2.15, 28.36), *P* = 0.002	7.58 (2.09, 27.58), *P* = 0.002	4.74 (1.27, 17.64), *P* = 0.02
Post vs. Pre in RYGB compared to Post vs. Pre in HR	3.43 (0.94, 12.56), *P* = 0.06	3.33 (0.91, 12.18), *P* = 0.07	2.05 (0.55, 7.65), *P* = 0.28

^a^Model covariates include age, sex, index admission length of stay, and Elixhauser comorbidities (minus obesity)

^b^Sensitivity analysis model covariates include age, sex, index admission length of stay, Elixhauser comorbidities (minus obesity), alcohol use, gallstones, and prior cholecystectomy

### Risk factors for AP after VSG and RYGB

We examined risk factors for AP after VSG and RYGB. The univariable analysis is described in Table 2S. The rates of alcohol use, gallstones, and prior cholecystectomy were similar between VSG and RYGB patients with pancreatitis (Table 2S). A multivariable analysis (Table 4) showed that an increased risk of AP within 6 months after VSG was associated with younger age (18–29 years, aOR 3.76, 95% CI: 1.68–8.45), female gender (aOR = 1.99; 95% CI: 1.04, 3.80), patients on Medicare (aOR = 2.50; 95% CI: 1.20, 5.19), and hospitalization for ≥ 3 days for VSG surgery (aOR = 3.53; 95% CI: 2.15, 5.77). Alternatively, self-pay patients were at lower risk of developing AP within 6 months after VSG (aOR = 0.14; 95% CI: 0.02, 0.97). For patients with RYGB, post-surgery AP was associated with younger age (18–29 years; aOR = 6.40; 95% CI: 2.49, 16.46). There was a significant interaction between AP and gallstones and prior cholecystectomy in VSG and RYGB patients. Patients with gallstones and no prior cholecystectomy had the highest AP risk compared to those with no history of either (VSG: aOR = 85.1; 95% CI: 52.4, 138.2; *P* < 0.001 and RYGB: aOR = 46.0; 95% CI: 24.3, 86.8; P < 0.001). Prior cholecystectomy trended towards a higher AP risk in both VSG and RYGB compared to the same reference irrespective of gallstone status, although the odds were much lower than for patients with gallstones and no prior cholecystectomy (Table 4).

**Table 4:** Multivariable logistic regression analysis for factors associated with AP admission within 6 months after VSG and RGB

Factors associated with AP risk within 6 months after VSG		
Variable	Odds ratio (95% CI)^a^	*p*-value^a^
Age range		0.005^b^
8–29	3.76 (1.68, 8.45)	0.001
30–49	1.87 (0.99, 3.56)	0.06
≥50	Reference	
Gender: Female vs. male	1.99 (1.04, 3.80)	0.04
Index length of stay, ≥3 vs. <3 days	3.53 (2.15, 5.77)	<0.001
Primary Payer		0.02^b^
Medicare	2.50 (1.20, 5.19)	0.01
Medicaid	0.72 (0.37, 1.38)	0.32
Self-pay	0.14 (0.02, 0.97)	0.047
Other	0.65 (0.09, 4.73)	0.67
Private Insurance	Reference	
Gallstones with no h/o cholecystectomy	85.1 (52.4, 138.2)	<0.001
H/o cholecystectomy with no gallstones	3.31 (1.37, 7.98)	0.01
H/o cholecystectomy with gallstones	2.54 (0.50, 13.02)	0.26
H/o of neither gallstones nor cholecystectomy	Reference	

Factors associated with AP risk within 6 months after RYGB		
Variable	Odds ratio (95% CI)	*p*-value
Age range		<0.001^b^
18–29	6.40 (2.49, 16.46)	0.001
30–49	3.04 (1.50, 6.15)	0.002
≥50	Reference	
Elixhauser Index (minus obesity)		0.07^b^
0	Reference	
1–2	0.64 (0.33, 1.26)	0.20
3–4	1.47 (0.68, 3.20)	0.33
≥5	1.51 (0.38, 6.09)	0.55
Gallstones with no h/o cholecystectomy	46.0 (24.3, 86.8)	<0.001
H/o cholecystectomy with no gallstones	1.38 (0.32, 6.01)	0.67
H/o Cholecystectomy with gallstones	2.60 (0.82, 8.27)	0.11
H/o of Neither Gallstones nor Cholecystectomy	Reference	

*H/O* history of

^a^Variables with *p* < 0.1 in univariable analyses were included in the multivariable model

^b^Omnibus *p*-value for variable (tests for overall differences among variable levels)

### Impact of RYGB, VSG, and HR on AP outcomes

AP after VSG and RYGB was mild without any end-organ damage or ICU admissions, and few required invasive interventions (Table 3S). Notably, 8.7% of VSG and <8% of RYGB patients had recurrent AP within 6 months after surgery. Cholecystectomy was performed on the same admission for gallstone AP in 60% of RYGB and 55.7% of VSG patients; however, it was performed infrequently prior to gallstone AP or afterwards.

## Discussion

The rates of AP have increased in previous decades, making it a major economic burden in the U.S^1,2^. Bariatric surgery is associated with increased risk of gallstone disease^22,23^. In this study, we compared the impact of VSG and RYGB on AP rates, risk factors, and outcomes. To our knowledge, this study is the largest and first to address this question using a surgical control. We included 652,042 morbidly obese patients who underwent RYGB, VSG, or controls (HR) from a database representative of the U.S. population. We demonstrated a 2-fold and a 7-fold post- vs. pre-surgery increase in AP risk in VSG compared to RYGB and HR, respectively. However, the increased AP risk after RYGB was not significant when compared to the HR controls. These results persisted after our sensitivity analysis adjusting for gallstones, alcohol use, and prior cholecystectomy. In a multivariable analysis model, gallstones were associated with increased AP risk after bariatric surgery and more so for VSG. Although no subjects had severe AP, the rates of same-admission cholecyscteomy was only around 55% for VSG patients with gallstone AP.

In this study, we compared the rates of AP within 6 months pre- and post-surgery in morbidly obese patients who underwent VSG, RYGB, and HR. As expected with morbid obesity, the rates of AP in our pre-surgery cohorts (0.04–0.17%) were higher than the estimates of AP in the general population^24,33^. Moreover, HR patients had the highest rate of AP pre-surgery, likely due to higher prevalence of alcohol use and gallstones compared to VSG and RYGB^24^. RYGB and VSG led to a steep rise in AP rates in the first 6 months after surgery, ranging between 0.17% and 0.21%, but not after HR. These rates are slightly lower than the previously reported range of 0.2–1.04%, probably because of different designs, small sample sizes, and variable follow up in prior studies^22,23,25,26,28–33^. VSG led to a higher pre- to post-surgery AP risk when compared to RYGB and HR after adjusting for multiple covariates. Conversely, RYGB was not associated with higher increase in risk of AP when compared to HR controls.

We then identified potential risk factors for AP after VSG and RYGB. Younger age was paradoxically associated with an increased risk of AP following both VSG and RYGB^24^. Gallstones in the absence of cholecystectomy were associated with an increased risk of AP after bariatric surgery, with a more striking impact in VSG. Prior cholecystectomy trended towards higher risk of AP after both VSG and RYGB, although AP risk was much less than gallstones in the absence of cholecystectomy. Other risk factors for AP after VSG included female gender that also suggests an underlying biliary etiology^45,46^. A prolonged hospital stay (≥3 days), previously linked to a higher risk of subsequent readmissions after bariatric surgery, was also associated with increased risk of AP after VSG in our study^47–49^. Medicare insurance was associated with higher risk of AP after VSG, while a self-pay status was associated with a lower risk of AP. Medicare beneficiaries undergoing bariatric surgery are mostly younger than 65 with disabilities and comorbidities and that may explain the increased risk of AP in this population^50^. Alternatively, self-pay status for bariatric surgery are more likely to suggest the financial ability to pay out of pocket and is usually associated with a lower risk of cholelithiasis^51,52^. These findings combined suggest that biliary disease is the main driver for the increased risk of AP after bariatric surgery, especially after VSG, and that prior cholecystectomy may reduce this risk. The stronger associated between AP and gallstones in VSG necessitates continued investigation. Potential explanation could be due to a larger postprandial peak of cholecystokinin (CCK) after VSG compared to RGB^53^. CCK promotes gallbladder contraction and the release of pancreatic enzymes and may be responsible for the higher risk of AP in VSG patients with gallstones compared to RYGB^54,55^. CCK may also explain the paradoxical association between AP and bariatric patients in the lower age groups due to higher sensitivity of the gallbladder to CCK in younger individual^56^. An alternative explanation could be skewed results due to the underrepresentation of symptomatic biliary disease (and as a result, gallstones diagnosis) in patients without pancreatitis in general and especially in VSG compared to RYGB. However, a prior study show similar rate of asymptomatic and symptomatic cholelithiasis when VSG was directly compared to RYGB, although data is limited^57^. Thus, further studies validating our findings and testing the correlation between the presence gallstones, CCK, and AP after VSG and RYGB are warranted.

Although AP risk increased after VSG, its rate was low at 21 per 1000 patients and does not warrant a prophylactic cholcyestectomy after every VSG. According to current bariatric surgery management guidelines, ultrasound measurements is conventionally utilized for the detection of gallstone formation in bariatric surgery patients, although a precise time frame is not clear^58^. In parallel, the prophylactic administration of ursodeoxycholic acid has been shown to reduce the risk of gallstone formation and symptomatic gallstone disease after bariatric surgery^59,60^. More than 50% of AP cases occurred within 30 days post-surgery; therefore, we recommend a selective strategy by performing ultrasonographic surveillance pre-surgery and 3 weeks after surgery in patients who fit the risk profile for AP (ages between 18 and 29 years old, females, Medicare insurance carriers and those with surgery admission ≥3 days) in order to detect gallstones. We then recommend discussing the risk:benefit ratio of performing cholecystectomy in patients with subsequent identification of gallstones. We also recommend better adherence to medical prophylaxis with ursodeoxycholic acid in patients fitting the risk profile for AP even if they have had a prior cholecystectomy. Future studies testing the utility of these clinical factors combined with novel serum biomarkers as better predictors of cholelithaisis and AP after VSG may also improve clinical decision making of cholecystectomy at the time of bariartric surgery.

The strength of our study lies in using the only U.S.-representative database that can track readmissions and obtain 6 months post-surgery AP rates. We used validated ICD-9CM codes and accounted for multiple confounders by using exclusions and adjustments. Some limitations include the retrospective design and the NRD’s reliance on ICD-9-CM coding, which makes it susceptible to bias due to coding and billing errors. There is also an inherent lack of specific factors such as surgeons’ expertise/technique, patient selection, and other potential confounders due to the nature of the NRD database. For instance, although the rates of diagnosed gallstones and cholecystectomies were similar between VSG and RYGB, we were unable to ascertain the rate of asymptomatic gallstone disease or compliance with ursodiol after bariatric surgery. While alcohol abuse may have been underestimated in our cohorts, other studies identify a lower risk of alcohol abuse in the immediate 6 months after bariatric surgery^61^. We were also unable to directly quantify baseline BMI, the amount of weight loss, triglycerides levels, or tobacco use post-surgery and their impact on AP risk. However, VSG leads to a lesser weight loss in the first 6 months compared to RYGB while HR is not associated with weight loss^40,62^. Furthermore, triglycerides are usually improved and the frequency of smoking is low and not changed after bariatric surgery^61,63^. Finally, it is possible that an AP attack may delay bariatric surgery leading to a lower pre-surgery AP rate, however the delay is not expected to be beyond 6 months from surgery since other surgeries like cholecystectomy can in fact be done safely and electively shortly after an AP admission. Furthermore, the inclusion of morbidly obese patients undergoing hernia repair as an additional control was meant to account for this potential bias. Finally, we could not include a follow up beyond 6 months since we had to divide each year equally to 6 months follow-up periods pre- and post-surgery in order to compare pre- to post-surgery rates of AP.

In conclusion, VSG is associated with increased AP risk compared to RYGB and control. Gallstones play a major role in increased AP risk after bariatric surgery, especially for VSG. Fortunately, AP presentation is usually mild. Furthermore, it is technically easier to decompress the bile ducts after VSG compared to RYGB. Our study emphasizes the need to adhere to current bariatric surgery guidelines of post-operative ursodiol utilization, as well as early ultrasonographic screening for gallstones, especially in women and patients younger than 50 years of age undergoing VSG. Further prospective studies validating our findings with mechanisms and targeted prevention strategies to decrease the risk of AP are warranted.

## Study Highlights

### What is current knowledge

- Acute pancreatitis is a leading cause of gastrointestinal hospital admissions in the U.S.
- Bariatric surgery is associated with an increased risk of gallstones disease.
- There are no previous studies investigating the differential impact of bariatric surgery type on risk of acute pancreatitis and none used a surgical control.

### What is new here

- This is the first national-level study looking at risk of AP after bariatric surgery.
- We document a 2-fold greater increase in acute pancreatitis risk after vertical sleeve gastrectomy when compared to roux-en-Y gastric bypass.
- The key risk factors for acute pancreatitis after bariatric surgery are younger age and presence of gallstones.

## Electronic supplementary material

Supplementary Tables

Supplementary Informations
