# Correction: The Type of Bariatric Surgery Impacts the Risk of Acute Pancreatitis: A Nationwide Study

**DOI:** 10.1038/s41424-018-0063-y

**Published:** 2018-10-08

**Authors:** Hisham Hussan, Emmanuel Ugbarugba, Kyle Porter, Sabrena Noria, Bradley Needleman, Steven K. Clinton, Darwin L. Conwell, Somashekar G. Krishna

**Affiliations:** 1 Obesity and Bariatric Endoscopy Section, Division of Gastroenterology, Hepatology, & Nutrition, Department of Internal Medicine, The Ohio State University Wexner Medical Center, Columbus, OH USA; 2 Division of Hospital Medicine, Department of Internal Medicine, The Ohio State University Wexner Medical Center, Columbus, OH USA; 3 Center for Biostatistics, Department of Biomedical Informatics, The Ohio State University, Columbus, OH USA; 4 Center for Minimally Invasive Surgery, Department of General Surgery, The Ohio State University Wexner Medical Center, Columbus, OH USA; 5 Division of Medical Oncology, Department of Internal Medicine, The Ohio State University Wexner Medical Center, Columbus, OH USA; 6 Division of Gastroenterology, Hepatology, & Nutrition, Department of Internal Medicine, The Ohio State University Wexner Medical Center, Columbus, OH USA; 7 Comprehensive Cancer Center, The Ohio State University, Columbus, OH USA

Correction to: *Clinical and Translational Gastroenterology*; 10.1038/s41424-018-0045-0; published online 12 September 2018

In the original version of this article, a footnote was missing. The PDF and HTML versions of the article have now been corrected to include the following:

This study was presented as an abstract at the World Congress of Gastroenterology (WCOG) at ACG 2017, Orlando, FL, USA.

