# Supplementary material for: The Type of Bariatric Surgery Impacts the Risk of Acute Pancreatitis: A Nationwide Study
Source: Clin Transl Gastroenterol. 2018 Sep 12;9(9):179. doi: 10.1038/s41424-018-0045-0 (PMC6134111; doi:10.1038/s41424-018-0045-0)
Supplement: Supplementary file 1 — Supplementary Tables [file 41424_2018_45_MOESM1_ESM.docx]

**Table 1S: Comparison of Individual comorbidities at time of surgery between VSG RYGB and HR patients**

| **Comorbidity** | **VSG (n=205,251)** | **RYGB (n=169,973)** | **HR (n=16,845)** |
| --- | --- | --- | --- |
| AIDS | 62 (0.0%) | 30 (0.0%) | * |
| Alcohol abuse | 543 (0.3%) | 462 (0.3%) | 116 (0.7%) |
| Deficiency anemias | 9,204 (4.5%) | 8,388 (4.9%) | 1,507 (8.9%) |
| Rheumatoid arthritis/collagen vascular diseases | 3,645 (1.8%) | 2,857 (1.7%) | 409 (2.4%) |
| Chronic blood loss anemia | 177 (0.1%) | 171 (0.1%) | 54 (0.3%) |
| Congestive heart failure | 3,037 (1.5%) | 3,426 (2.0%) | 596 (3.5%) |
| Chronic pulmonary disease | 36,659 (17.9%) | 34,945 (20.6%) | 3,855 (22.9%) |
| Coagulopathy | 908 (0.4%) | 803 (0.5%) | 216 (1.3%) |
| Depression | 41,472 (20.2%) | 42,904 (25.2%) | 3,219 (19.1%) |
| Diabetes, uncomplicated | 49,506 (24.1%) | 59,585 (35.1%) | 4,124 (24.5%) |
| Diabetes with chronic complications | 3,798 (1.9%) | 5,347 (3.1%) | 501 (3.0%) |
| Drug abuse | 741 (0.4%) | 649 (0.4%) | 142 (0.8%) |
| Hypertension (combine uncomplicated and complicated) | 108,244 (52.7%) | 101,645 (59.8%) | 9,918 (58.9%) |
| Hypothyroidism | 24,165 (11.8%) | 21,628 (12.7%) | 2,470 (14.7%) |
| Liver disease | 23,003 (11.2%) | 25,098 (14.8%) | 1,011 (6.0%) |
| Lymphoma | 176 (0.1%) | 113 (0.1%) | 14 (0.1%) |
| Fluid and electrolyte disorders | 5,020 (2.4%) | 5,326 (3.1%) | 1,222 (7.3%) |
| History of metastatic cancer | 36 (0.0%) | 18 (0.0%) | 34 (0.2%) |
| Other neurological disorders | 4,245 (2.1%) | 4,499 (2.6%) | 626 (3.7%) |
| Paralysis | 249 (0.1%) | 175 (0.1%) | 53 (0.3%) |
| Peripheral vascular disorders | 1,206 (0.6%) | 1,415 (0.8%) | 268 (1.6%) |
| Psychoses | 6,045 (2.9%) | 6,472 (3.8%) | 567 (3.4%) |
| Pulmonary circulation disorders | 1,049 (0.5%) | 1,165 (0.7%) | 225 (1.3%) |
| Renal failure | 3,757 (1.8%) | 3,668 (2.2%) | 727 (4.3%) |
| Solid tumor without metastasis | 207 (0.1%) | 171 (0.1%) | 55 (0.3%) |
| Peptic ulcer disease excluding bleeding | 15 (0.0%) | 24 (0.0%) | * |
| Valvular disease | 1,897 (0.9%) | 1,428 (0.8%) | 372 (2.2%) |
| Weight loss | 162 (0.1%) | 314 (0.2%) | 167 (1.0%) |

* The cell’s value is not displayed. As per data agreements with AHRQ, researchers cannot report any statistics where the number of observations in any given cell of analyzed data is ≤ 10.

**Table 2S: Univariable logistic regression analysis for factors associated with AP admission within 6 months after VSG and RYGB**

|  | **VSG** | | **RYGB** | |
| --- | --- | --- | --- | --- |
| **Variable** | **Odds ratio (95% CI)** | **p-value** | **Odds ratio (95% CI)** | **p-value** |
| Age range |  | **0.02*** |  | **<0.001*** |
| 18–29 | 2.56 (1.32, 4.94) | 0.01 | 5.18 (2.26, 11.88) | <0.001 |
| 30–49 | 1.34 (0.78, 2.31) | 0.30 | 2.58 (1.28, 5.17) | 0.01 |
| ≥50 | reference | | | |
|  |  |  |  |  |
| Gender: Female vs. Male | 2.10 (1.12, 3.94) | **0.02** | 1.70 (0.86, 3.34) | 0.13 |
|  |  |  |  |  |
| Index Length of Stay: ≥3 vs. <3 days | 3.74 (2.33, 6.01) | **<0.001** | 1.10 (0.61, 1.98) | 0.75 |
|  |  |  |  |  |
| Elixhauser Index (minus obesity) |  | 0.61* |  | **0.09*** |
| 0 | reference | | | |
| 1–2 | 0.84 (0.51, 1.37) | 0.48 | 0.46 (0.24, 0.89) | 0.02 |
| 3–4 | 1.21 (0.66, 2.21) | 0.54 | 0.83 (0.42, 1.67) | 0.61 |
| ≥ 5 | 0.64 (0.15, 2.72) | 0.55 | 0.77 (0.22, 2.74) | 0.69 |
|  |  |  |  |  |
| Primary Payor |  | **0.03*** |  | 0.19* |
| Medicare | 1.83 (1.01, 3.30) | 0.047 | 1.90 (1.04, 3.47) | 0.04 |
| Medicaid | 0.95 (0.51, 1.78) | 0.88 | 1.45 (0.73, 2.85) | 0.29 |
| Private Insurance | reference | | | |
| Self-pay | 0.10 (0.01, 0.71) | 0.02 | 0.40 (0.05, 2.89) | 0.36 |
| Other | 0.55 (0.07, 4.00) | 0.55 | 1.12 (0.33, 3.76) | 0.86 |
|  |  |  |  |  |
| Income Quartile |  | 0.11* |  | 0.51* |
| Quartile 1 | reference | | | |
| Quartile 2 | 0.51 (0.26, 1.03) | 0.06 | 0.83 (0.38, 1.80) | 0.64 |
| Quartile 3 | 0.89 (0.50, 1.59) | 0.70 | 1.33 (0.67, 2.65) | 0.42 |
| Quartile 4 | 0.52 (0.28, 0.97) | 0.04 | 0.88 (0.40, 1.92) | 0.74 |
| Missing data | 0.31 (0.04, 2.24) | 0.24 | n/a^ | n/a^ |
|  |  |  |  |  |
| Hospital Type |  | 0.69* |  | 0.93* |
| Urban non-teaching | reference | | | |
| Urban teaching | 1.06 (0.66, 1.72) | 0.80 | 1.10 (0.67, 1.79) | 0.71 |
| Rural | 1.61 (0.55, 4.74) | 0.39 | 0.99 (0.15, 6.33) | 0.99 |
|  |  |  |  |  |
| Hospital Bed-size |  | 0.50* |  | 0.42* |
| Small | reference | reference | reference | reference |
| Medium | 0.69 (0.36, 1.32) | 0.26 | 0.64 (0.31, 1.31) | 0.22 |
| Large | 0.74 (0.41, 1.34) | 0.32 | 0.70 (0.37, 1.30) | 0.26 |
|  |  |  |  |  |
| Alcohol use (yes) | n/a^ | n/a^ | n/a^ | n/a^ |
| Gallstones (yes) | 19.74 (12.36, 31.54) | **<0.001** | 13.33 (7.63, 23.30) | **<0.001** |
| Prior cholecystectomy (yes) | 2.16 (0.93, 5.00) | **0.07** | 1.32 (0.53, 3.27) | 0.55 |
| Surgical complications (yes) | n/a^ | n/a^ | 2.14 (0.81, 5.62) | 0.12 |

* Omnibus p-value for variable (tests for overall differences among variable levels)

^ 0 cases of pancreatitis in the alcohol use (both), missing income (RYGB), and surgical complications (VSG) groups

^$^ Age and length of stay were not linearly associated with the log-odds ratio and therefore were included in the model as categorical variables rather than continuous.

**Table 3S: Post-surgery AP outcomes or interventions**

| **Outcomes at AP admission** | **RYGB (n=138 AP)** | **VSG (n=196 AP)** |
| --- | --- | --- |
| Severe pancreatitis | 0 | 0 |
| Mechanical ventilation | 0 | 0 |
| Hemodynamic shock | 0 | 0 |
| Hemodialysis | 0 | 0 |
| Sepsis | * | 0 |
| Intra-abdominal infection | 0 | 0 |
| Recurrent pancreatitis within 6 month study period | * | 17 (8.7%) |
|  |  |  |
| **Interventions at AP admission** |  |  |
| ERCP | * | * |
| Open gastrostomy tube | * | 0 |
| Percutaneous biliary procedures | 0 | 0 |
| Open biliary procedures | 0 | 0 |
| Any surgical pancreatic procedure related to AP event | 0 | 0 |
|  |  |  |
| Gallstone AP | 50 (36.2%) | 70 (35.7%) |
| Cholecystectomy prior to Gallstone AP admission | * | * |
| Cholecystectomy at Gallstone AP admission | 30 (60%) | 39 (55.7%) |
| Cholecystectomy after Gallstone AP admission, until 6 months after surgery | * | * |

*The cell’s value is not displayed. As per data agreements with AHRQ, researchers cannot report any statistics where the number of observations in any given cell of analyzed data is ≤ 10.

**Supplemental Table 4S: ICD-9-CM Codes used in the Study:**

| **Diagnosis/Procedure** | **ICD-9-CM codes** | **Variable location** |
| --- | --- | --- |
| Obesity | 278.01, V85.35, V85.36, V85.37, V85.38, V85.39, or V85.4X | DX1-DX30 |
| Laparoscopic Roux-en-Y gastric bypass surgery | 44.38 | PR1-PR15 |
| Laparoscopic Vertical sleeve gastrectomy | 43.82 | PR1-PR15 |
| Laparoscopic ventral or umbilical hernia repair  (without mention of obstruction or gangrene) | 553.1, 553.29, 553.20, 553.21 plus either 53.42, 53.43, 53.62, 53.63 | DX1-30 + PR1-PR15 |
| Laparoscopic inguinal hernia repair (without mention of obstruction or gangrene) | 550.9X plus either 17.11, 17.12, 17.13, 17.21, 17.22, 17.23, 17.24 | DX1-30 + PR1-PR15 |
| Laparoscopic diaphragmatic hernia repair (without mention of obstruction or gangrene) | 553.3 plus either 53.83, 53.71 | DX1-30 + PR1-PR15 |
| Exclusions: |  |  |
| Pregnancy status | DX 792.3, 796.5, V61.7, V65.11, any diagnosis code starting with 63, 64, 65, 66, 670, 671, 672, 673, 674, 675,676, 677, V22, V24,V27,V28.0,282, V286,V616,V617  PR 75.3, 75.30, 75.31, 75.32, 75.33, 75.34, 75.35, 75.36, 75.37, any procedure code staring with 72, 73, 74, 75.0, 75.1, 75.2, 75.4, 75.5 | DX1-DX30  PR1-PR15 |
| Abdominal neoplasm | Any code starting with 150.0 through 159.9 | DX1-DX30 |
| History of chronic Pancreatitis | 577.1 | DX1-DX30 |
| Pancreatic Cyst | 577.2 | DX1-DX30 |
| History of bariatric surgery | V45.86 | DX1-DX30 |
| Open surgery | 44.31,44.39, 53.61 or 53.69, 53.0x, 53.1x, 53.51, 53.59, 53.49, 53.41, 53.84, 53.72 | PR1-PR15 |
| Gangrene, obstruction of hernia | 551.x, 552.x, 550.0, 550.1 | DX1-30 |
| Outcome: |  |  |
| Acute Pancreatitis | 577.0 | DX1 |
| Etiologies: |  |  |
| Biliary disease: Cholelithiasis or Choledocholithiasis | 574, 574.00, 574.01, 574.10, 574.11, 574.20, 574.21, 574.30, 574.31, 574.40, 574.41, 574.50, 574.51, 574.60, 574.61, 574.70, 574.71, 574.80, 57.81, 574.90, 574.91 | DX1-DX30 |
| Alcohol | 291.0, 291.1, 291.2, 291.3, 291.4, 291.5, 291.81, 291.82, 291.89, 291.9, 303.00, 303.01, 303.02, 303.03, 303.90, 303.91, 303.92, 303.93, 305.00, 305.01, 305.02, 305.03, 760.71, 980.0, 357.5, 425.5, 535.30, 535.31, 571.0, 571.1, 571.2, 571.3 | DX1-DX30 |
| Complication of GI procedure | 997.4 | DX1-DX30 |
| Cholecystectomy | 51.21, 51.22, 51.23, 51.24 | PR1-PR15 |
| Cholecystectomy, history of | V45.79 | DX1-DX30 |
| Interventions at the time of pancreatitis admission |  |  |
| ERCP | 51.83, 51.84, 51.85, 51.86, 51.87, 51.88, 51.10, 51.11, 51.14, 52.13,  52.14, 52.93, 52.94, 52.97, 52.98, 97.05 | PR1-PR15 |
| Open gastrostomy tube | 43.19 | PR1-PR15 |
| Percutaneous biliary procedures | 51.01, 51.96, 51.98 | PR1-PR15 |
| Open biliary procedures (common bile duct exploration) | 51.02, 51.03, 51.04, 51.32, 51.36, 51.37, 51.39, 51.41, 51.43, 51.51, 51.59, 51.63, 51.64, 51.69, 51.71, 51.79 | PR1-PR15 |
| Any surgical pancreatic procedure related to AP event | 52.01, 52.09, 52.22, 52.51, 52.52, 54.91, 54.0, 54.11, 54.19, 54.99, 31.2 | PR1-PR15 |
| Acute Respiratory failure and mechanical ventilation | 93.90, 96.01, 96.02, 96.03, 96.04, 96.05, 96.70, 96.71, 96.72 | PR1 to PR15 |
|  | 518.0, 518.81, 518.82, 518.84 | DX2-DX30 |
| Cardiac failure (surrogate: Infusion of intravenous vasopressin) | 00.17 | PR1 to PR15 |
| Acute kidney injury and hemodialysis | 39.95 | PR1-PR15 |
|  | 584.5, 584.6, 584.7, 584.8, 584.9, 586 | DX2-DX30 |
| Intra-abdominal infections | 567.2, 567.1, 567.29, 567.22, 567.38, 567.39 | DX2-DX30 |
| Sepsis | 785.52, 03.8X, 995.9 | DX2-DX30 |
